# Early Social Stress Promotes Inflammation and Disease Risk in Rhesus Monkeys

**DOI:** 10.1038/s41598-019-43750-1

**Published:** 2019-05-20

**Authors:** Erin L. Kinnally, Steten J. Martinez, Katie Chun, John P. Capitanio, Lesly C. Ceniceros

**Affiliations:** 1 University of California Davis, Department of Psychology, Davis, USA; 2 California National Primate Research Center, Davis, USA

**Keywords:** Psychology, Gastrointestinal diseases

## Abstract

Early social stress has potent lifelong health effects. We examined the association of early stress in the attachment relationship (low maternal sensitivity, low MS), lower maternal social hierarchy rank, and greater frequency of group-level social conflict, with biomarkers of inflammatory stress response in plasma (IL-8, MCP-1 and CRP collected two hours after temporary separation from mothers and social groups) and risk for developing a common macaques disease outcome (infectious colitis) in 170 socially-housed rhesus monkeys. We controlled for gene-environment correlations by comparing cross-fostered subjects with infants reared by their biological mothers. Low MS predicted higher levels of pro-inflammatory cytokines and proteins at 3–4 months of age (F(3, 162) = 3.508, p = 0.002, partial eta^2^ = 0.061) and higher lifetime risk for developing colitis for up to twelve years of age (chi square = 5.919, p = 0.026). Lower maternal social rank (F (3, 162) = 3.789, p = 0.012, partial eta^2^ = 0.06) and higher rates of social conflict (F (3, 162) = 4.264, p = 0.006, partial eta^2^ = 0.074) each also predicted greater inflammation in infancy, but not lifetime colitis risk (both p > 0.05). The effects of low MS, lower social rank, and higher social conflict were significant in infants reared by biological mothers and cross-fostered infants, suggesting that our results did not arise from gene-environment correlations, but environmental stressors alone. We conclude that several types of early social stress confer risk for inflammation in infancy, but that stress in the mother-infant relationship may confer the longest-term risk for adverse health outcomes.

## Introduction

Early adversity is one of the most potent psychosocial predictors of disease and mortality in humans^1–5^. One mechanism for this risk is the re-orchestration of communication between the stress response and immune systems^2,3^. For example, chronic inflammation has been repeatedly shown to increase risk for disease^6–11^. Though inflammation is an essential immune function, necessary to recover from infection or trauma, chronically elevated levels of pro-inflammatory cytokines desensitizes the immune system and remodels tissue to promote infectious, neurodegenerative, metabolic and cardiovascular disease^9–11^.

In humans, there is extensive evidence that multiple factors, such as low socioeconomic status and poverty^6^, childhood sexual abuse^12^, physical abuse^13,14^, neglect^15^, and poor parental care^16^ are all risk factors for inflammation and poor health outcomes in children. Stress within the attachment relationship may be particularly formative. Disruption of attachment relationships (ie, separation from parents) has repeatedly been shown to shift health trajectories in animal models^17–19^. Particularly vital, however, is the study of the consequences of stress within intact attachment relationships. These nuanced experiences are the primary elements of most children’s psychosocial environment. Good quality parental care can ameliorate the effects of the harshest life stressors^20–23^, and poor quality care can put a child at a disadvantage from the earliest stages of life, even when other resources are available^24–26^. One of the most powerful measures of parental care in humans is Mary Ainsworth’s maternal sensitivity (MS) scale^27^. MS includes four dimensions of responsiveness to an infant: sensitivity/insensitivity, acceptance/rejection, availability/neglect and cooperation/interference^28–30^. Sensitive mothers respond promptly and empathetically toward their infants, and are more available, less rejecting, and more cooperative with the infant than less sensitive mothers. Low MS is presumed to be stressful for infants because the primary attachment figure is not always available for prompt, empathetic, and mutualistic interactions with the child. This is a useful index for maternal care because it depends on the unique needs of the child, rather than assigning common types (or rates) of maternal behavior as “good” or “bad” quality. For example, a protective response is only stress-buffering if the child is afraid: to a relaxed child, a protective response may be unexpected and stressful. A neglecting response is only neglectful if the infant desires attention^28–30^. Low MS may occur for many reasons, including maternal inexperience, stress, or depression, but even controlling for these factors, the health inequities between low and high MS children are apparent early on: lower MS has been linked with failure to thrive^25^, growth abnormalities^31–33^, cardiovascular irregularities^34^ and inflammatory disease in childhood^20^. However, it is challenging to understand the true effects of low MS in humans in part because family dynamics are arise from interacting genetic and environmental factors^35,36^.

Rhesus monkeys (*Macaca mulatta*) are a highly translational animal model to prospectively track the effects of social stress on health without genetic confounds^37,38^. Rhesus monkeys exhibit comparable physiological, immune, and behavioral development to humans^39,40^. Additionally, infants can be fostered onto biologically unrelated mothers on the first day of life, providing an experimental disentanglement of correlated gene-environment influences on offspring^41^. We also incorporate a naturally- occurring facet of macaque maternal care that shares some features with human MS^18,42^. We examined the association of low MS with inflammation in infancy (3–4 months of age) using a cross-fostering design. We simultaneously evaluated the role of two other potential social stressors, maternal social hierarchy rank and rate of social conflict between social group members during early postnatal development. We hypothesized that low MS, lower maternal social rank, and higher rates of social conflict would each predict higher inflammation in infant macaques. Further, if the effects of social stressors were due to the stressors themselves rather than gene-environment correlations, we predicted that the effects of stress would be comparable between cross-fostered and infants reared by biological mothers. Finally, we then followed these subjects for up to twelve years of life, and examined risk for inflammatory disease over the lifespan to identify whether different elements of early social stress influenced lifelong disease risk.

## Methods

### Subjects

Subjects included 170 healthy infant rhesus macaques (90 female, 80 male). Subjects were either raised by their biological mothers (BIO, n = 132) or cross-fostered to a new unrelated female in a new social group (FOSTER, n = 38). These subjects represent a subset of subjects from a larger longitudinal study of 206 mother-infant pairs. All mother-offspring pairs were housed in the outdoor breeding colony at the California National Primate Research Center (CNPRC). Animals were housed in one of sixteen established social groups (30–160 members). These groups were comprised of similar social demographics, including 6–13 distinct matrilines with extended kin networks and animals of all age/sex classes. All social groups included 5–10 reproductively mature males, 23–105 reproductively mature females, and 25–75 subadult, juvenile or infant monkeys. Enclosures were 0.2 hectares with chain link fencing to allow visual access. Animals were fed monkey chow (LabDiet 5405) twice per day, once in the morning and once in the afternoon. All procedures were approved by the UC Davis Animal Care and Use Committee and were in compliance with the National Institutes of Health guide for the care and use of Laboratory animals.

### Cross-fostering procedures

As infants were born, they were assigned to BIO or FOSTER conditions. BIO dyads remained in large social groups for the first twelve weeks of life. Pairs of dyads to be cross-fostered were removed from outdoor enclosures on the day of infant birth. During indoor FOSTER procedures, infants were removed from the sedated (10 mg/kg ketamine) biological mothers by trained CNPRC staff and then placed on the ventrum of the sedated FOSTER mother that had recently given birth, FOSTER pairs were observed indoors for 18–24 hours by CNPRC staff for successful fostering/reunion. Within 24 hours of FOSTER manipulations, FOSTER dyads were returned to field enclosures until infants were 3–4 months of age. Cross fostering was conducted on only one pair per day.

### Social hierarchy rank determination

In nature and captivity, group living rhesus macaques form social hierarchies, in which each social group member occupies a rank-ordered place compared with all other group members. Hierarchy rank of the biological and foster mothers was measured by trained CNPRC Behavioral Management staff. Infant subjects are not considered to have social rank, as they are not yet independent from mothers. Hierarchy data were collected in all outdoor field corrals for at least 30 minutes on a bi-weekly basis, totaling at least one hour of data collection per month. Data were collected with scan sampling to record dyadic displacement interactions between individuals. Displacement interactions include aggression (chasing, biting, threats) from one individual, and subordination (fear grimace, moving out of proximity) from the other. The number of “wins” (when an individual receives subordination in response to an aggression) and losses (the same individuals subordinates in response to an aggression) are plotted against all other adult females in the group. Animals with the most wins and fewest losses are considered the highest ranked, and the animals with the most losses and fewest wins are considered the lowest ranked. We calculated maternal social rank as the absolute rank at the time of infant birth divided by the number of adult females in the social group (continuous variable). Ranks ranged from the 2% percentile for higher-ranked animals (i.e., 98% of female adults and adolescents are lower-ranked than mother) to the 100th percentile (i.e., lower ranked animals: 0% of adults and adolescents are lower ranked than mother) The average rank was 47.4%, such that 52.6% of animals were lower-ranked than our subjects’ mothers.

### Mother-infant observations

Mother-infant interactions were assessed and we present the detailed methods as we have described previously^18,40^. Dyads were observed in their social groups for five minutes per observation using focal dyadic sampling conducted one to four times weekly. Observations were conducted between 0700 h and 1300 h, during postnatal weeks 1–12. Observation order was rotated daily so dyads were observed at different times of day across the proscribed observation period.

Mother-infant interactions were coded using a transactional coding system, describing the overall theme of an interaction from the perspectives of the initiator and the recipient^18,40^. A transaction was defined as a change from one state of association that lasted three seconds or more, to a new state that was maintained for at least three seconds. Themes are defined in Table 1, and include protection, affiliation, neutral, rejection and aggression. Each dyadic interaction was characterized from the perspective of the mother and of the infant, and a theme assigned for each based on their behavior. The initiator of the transaction is considered to be the individual who changed the state of interaction between the pair, which the receiver then responds to. For example, common types of transaction types include: (1) infant approaches mother and initiates contact, mothers initiates physical contact (which would be scored as Affiliative-Affiliative, initiated by the infant), (2) Mother retrieves infant and infant grabs mother’s ventrum or back (Protective-Affiliative), initiated by the mother, (3) Infant jumped on mother, mother swats the infant to the ground (Affiliative-aggressive, initiated by the infant).

**Table 1:** Transaction Theme Definitions.

Protective (PRR)	Includes any action to physically control or limit the infant’s movement
Affiliative (AF)	Physical contact that is pro-social, including grooming, mounting, play, protection seeking, approach and clinging, food sharing, mouth-mouth contact, and nursing
Accommodate (ACC)	Any changing of physical position to accommodate a social overture without pro-social contact. For example, stopping to wait for the partner, huddling, presenting for grooming.
Neutral (N)	Initiating or maintaining proximity without pro-social contact or changed body position
Resist (RE)	Leaving proximity upon approach of partner, struggling to get away if they attempt to limit movement
Aggress (AG)	Antisocial contact including biting, hitting, scratching, flattening or dragging.

Male and female adult raters were trained by a primatologist with experience in mother-infant interactions for at least eight two-hour training sessions. Rater reliability was determined at the end of this period by calculating whether the rater was correct in recording each aspect of the transaction (maternal theme, infant theme, and recipient) for ten sequential five-minute trials. These trials were required to include with a wide variety of possible transaction calls or they were excluded from reliability calculations. The criterion for inter-rater reliability was 90% or better, and averaged 94% across all observers. Rates of all transaction themes by the mother (Protective, Affiliative, Accommodating, Neutral, Rejecting or Aggressive) were calculated per observation period for analysis.

### Maternal sensitivity scale

Our maternal sensitivity measure was characterized from factor analysis of 206 mother-infant dyads that were part of a longitudinal ongoing study. We present the methods here as we have described previously^18,40^.Transactions in which the infant initiated social overtures toward that mother were considered in this analysis. Rates of each transaction response type by the mother (continuous variables) were entered into a principal components analysis with Promax rotation. The analysis yielded five factors. We identified a factor that explained a high proportion of the total variance explained in maternal responses (17% out of 67%). This factor was novel in that it did not fit into the remaining factor structure which described factors that specifically overlapped with our other maternal behavior themes (ie, protectiveness, affiliation, aggressiveness and neutral; Kinnally *et al*.^18,40^). This factor was considered to be a naturally occurring aspect of macaque maternal care that shares similarities with Ainsworth’s MS scale: high MS mothers are more accepting and symmetrical in response to infants than low MS mothers, as in humans^18,27–29^ Maternal sensitivity scores were generated based on all factor loadings using the regression method. Higher MS scores reflect higher rates of affiliative or neutral responses to infant neutral and affiliative overtures (See Table 2 for factor loadings, bolded loadings over 0.4 are considered to represent significant contributors to factors).

**Table 2:** Factor Analysis for Maternal Sensitivity Score.

Infant (Initiator)	Mother (Responder)	Factor Loading
Affiliate	Protect	−0.126
Affiliate	Affiliate	**0.604**
Affiliate	Accomodate	−0.189
Affiliate	Neutral	**0.857**
Affiliate	Resist	0.075
Affiliate	Aggress	0.090
Neutral	Protect	−0.151
Neutral	Affiliate	−0.009
Neutral	Neutral	**0.751**
Neutral	Resist	0.028
Neutral	Aggress	0.075
Aggress	Aggress	0.128

### Social climate measures

Social climate was recorded at the end of every mother-infant observation. The social climate scale included five descriptors of the activity status of proportions of the social group, where activity was defined as locomotion, eating, drinking, or other activities in which the animal’s body was in motion. “Torpor” was recorded when less than 10% of the social groups members were engaged in activity, “Calm” was recorded when 10–25% of the group was active, “Moderately Active” was recorded for 25–50%, and “Active was recorded when 50–100% of groups members were engaged in activity. “Conflict” was recorded if two or more members of the social group engaged in contact aggression at any time during the observation. Another requirement was that 10% or more of the group must attend to conflict or change their behavior in response to conflict for conflict to be scored. The criterion for observer reliability for this measure was 90% or better, and across all observers there was an average of 95% reliability. For each dyad, a proportion of observations in which each social climate state was recorded was generated. Rate of social conflict ranged from 0% per observations to 39% per total observations per subject, with a mean of 11.4%.

### Cytokine and protein assays

At 3–4 months (90–120 days) of age, infants underwent a 25-hr social and maternal separation as part of a standardized biobehavioral assessment at the CNPRC. Inflammatory stress response was measured by assaying plasma cytokines and proteins (Interleukin 8 [IL-8], monocyte chemoattractant protein 1 [MCP-1] and C-reactive protein [CRP]) collected 2 hours after a mother-infant separation. Samples were assayed using the MILLIPLEX MAP Non-Human Primate Cytokine Magnetic Bead Panel Array Kit (Millipore, Billerica, MA, USA). Samples were assayed following manufacturer’s instructions. While the panel included a larger number of possible analytes (including IL-6 and TNFα), pilot testing revealed that reliability standards met our stringent criteria only for IL-8 and MCP-1 (ie, >99% standard curve fit and ~20% intra-assay coefficient of variance). Plates were read using a Bio-Plex HTF System with Luminex xMAP Technology (Bio-Rad, Hercules, CA, USA), and values were calculated using Bio-Plex Manager Software, version 4.1. Samples were run in duplicate, and duplicates with coefficients of variance less than 30% were included for analysis (average intra-assay CVs: IL-8 = 7.15% and MCP-1 = 8.16%). To control for inter-assay variation, a pooled sample was run on each plate. Interassay coefficients of variance were: MCP1 average = 18.05%; IL-8 average = 22.19%). Assay sensitivity for MCP-1 was 3.1 pg/ml and for IL-8 1.1 pg/ml. Observed ranges and means were: IL-8 (0.00–5131.06; 437.98 pg/ml) and MCP-1 (30.14–632–0.44; 213.28 pg/ml). A total of 36 out of our original 206 subjects were removed from the study because of missing or incomplete data.

C-reactive protein was measured using a latex agglutination method^43^. Latex particles are coated with rabbit anti-CRP antibodies and incubated with plasma from each subject. Displacement reactions are read using a Beckman Coulter AU 480 chemistry system (Beckman Coulter, Brea, CA). Observed CRP ranged from 0–33 pg/ml, with an average of 1.81 pg/ml.

### Health follow-up

Approximately 145 months (up to 12 years) later, we extracted health data from our subjects’ lifetime veterinary health records as we have described previously^39^. We recorded presence or absence of the most common infectious disease in captive macaques, colitis/diarrhea, at any time in the intervening years between assessment and the present, up to 12 years. These were defined as (1) having been hospitalized for one of these conditions, and (2) testing positive for one or more enteric pathogens that cause diarrhea including *Shigella, Campylobacter lari, Campylobacter coli, Campylobacter jejuni, orTrichomonas*. Of our subjects, 47% fit these criteria.

### Data analysis

All data analysis was conducted using SPSS version 26. The effects of MS (independent variable) on three plasma pro-inflammatory measures (dependent variables) was assessed using multivariate analysis of variance (MANOVA). This method was selected because it allows us to assess the effect of our predictors on correlated outcome measures (interclass correlation coefficient among cytokines = 0.432, p < 0.0001) and enhances power. Maternal sensitivity groups were trichotomized as one standard deviation or more above (high MS, n = 24), within one standard deviation (medium MS, n = 123), and one standard deviation or more below (low MS, n = 23) the population mean. MS group and foster condition (FOSTER or BIO rearing) were independent variables. Maternal social hierarchy rank (continuous variable), average proportion of observations containing social conflict across the dyad observation period (continuous variable), foster status (dichotomous variable), and infant sex (dichotomous variable), were entered as covariates. Earlier models included group level covariates (group identity and group size), but these were non-significant and therefore removed from the analysis. To test the specificity of association with MS, we included our other maternal behavior factors (protectiveness, affiliation, neutrality and aggression,^18^) in earlier models, but they were non-significant and therefore removed from the analysis. Interactions between infant sex, foster condition, maternal rank and social group conflict and MS were tested in separate MANOVAs. We used this stepwise approach to testing interactions because our study was not powered to test all interactions in the same model (ie, some cell sizes were too small to permit analysis). Two-tailed statistical significance was set at p < 0.05.

The effects of MS, foster status, maternal social hierarchy rank and social conflict on risk for lifetime inflammatory disease was analyzed using multinomial logistic regression with likelihood ratio testing. Non-significant contributors were manually removed from the model. For this analysis, one-tailed statistical significance was set at p < 0.05. One-tailed testing was used because our hypothesis was directional based on our previous study showing a link between poor maternal care and greater rates of colitis/diarrhea^40^. Finally, we determined whether inflammation in infancy predicted lifetime disease risk using independent samples t-tests.

## Results

Maternal Sensitivity (MS) did not differ between FOSTER and BIO mothers, (chi square = 0.064 p = 0.968), by rank (F(2, 169 = 2.190, p = 0.115), social conflict rates (F = 1.562, p = 0.212) or infant sex (chi-square = 5.322, p = 0.070).

**Figure 1:**
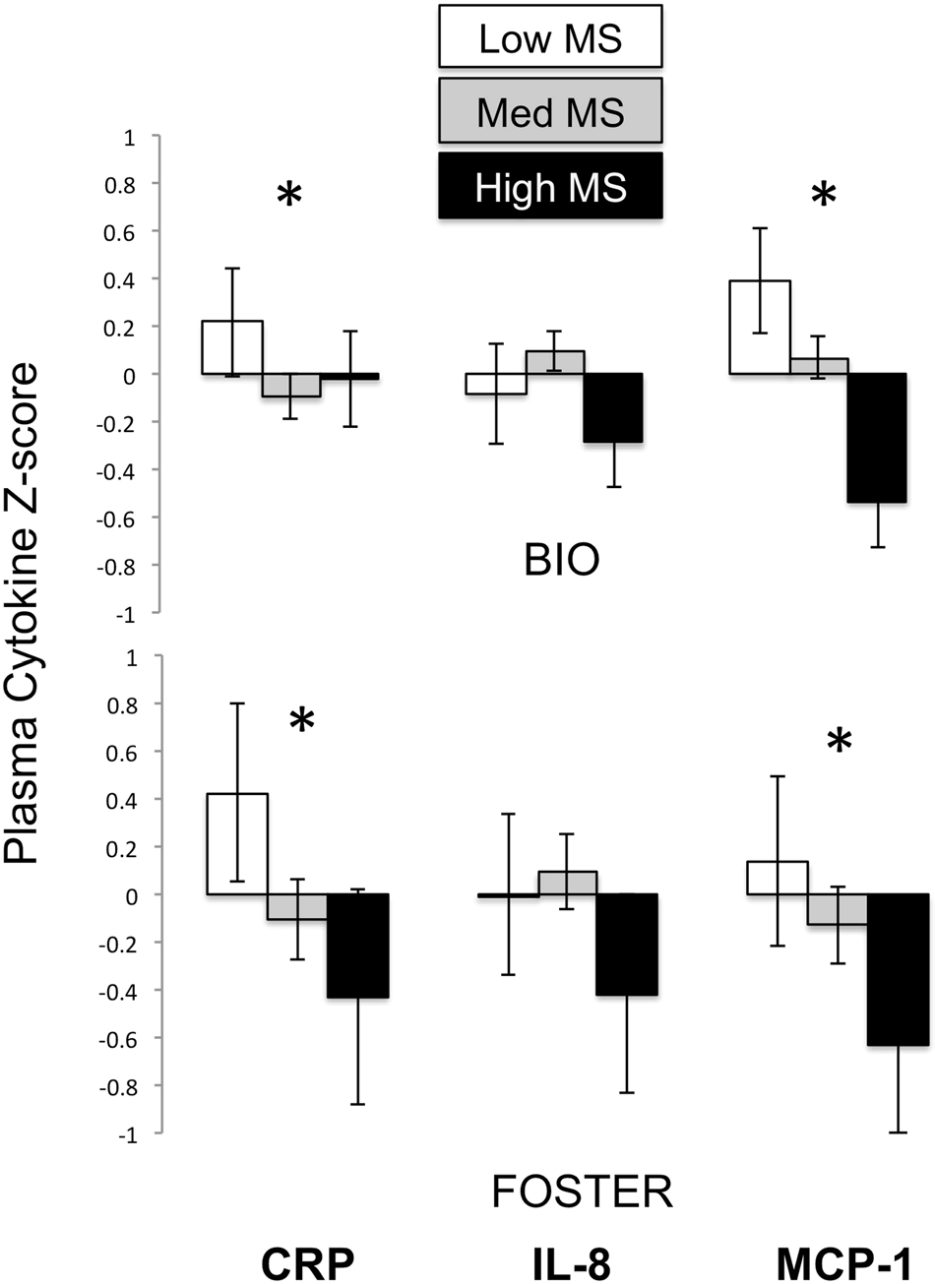
Low MS is associated with greater concentrations of pro-inflammatory cytokines and proteins in infants reared by biological mothers (BIO) and cross-fostered (FOSTER) infants. Means are presented +/− standard error of the mean. *p < 0.05.

Low MS was associated with significantly higher concentrations of inflammation biomarkers in infancy (F(3, 162) = 3.508, p = 0.002, partial eta^2^ = 0.061; Fig. 1). The effect was such that low MS was linked with significantly higher IL-8 (F(2, 163) = 3.213, p = 0.043), higher MCP-1 (F(2, 163) = 5.210, p = 0.006), and a trend for higher CRP ((F(2, 163) = 2.862, p = 0.060).

Foster status (F (3, 162) = 0.684, p = 0.563, partial eta^2^ = 0.013), and infant sex (F (3, 162) = 0.508, p = 0.667, partial eta^2^ = 0.009) did not significantly predict inflammation. Maternal social rank (F (3, 162) = 3.789, p = 0.012, partial eta^2^ = 0.06) and rate of social conflict (F (3, 162) = 4.264, p = 0.006, partial eta^2^ = 0.074) each predicted higher overall biomarkers of inflammation. Lower maternal rank predicted higher MCP-1 ((F(2, 163) = 5.771, p = 0.005) and higher CRP at the trend level ((F(2, 163) = 3.533, p = 0.060). Higher social conflict predicted higher CRP (F(2, 163) = 7.368, p = 0.007). There were no significant two-way interactions between MS and foster status, rank or social conflict that predicted inflammation (all p > 0.05; Fig. 1).

**Figure 2:**
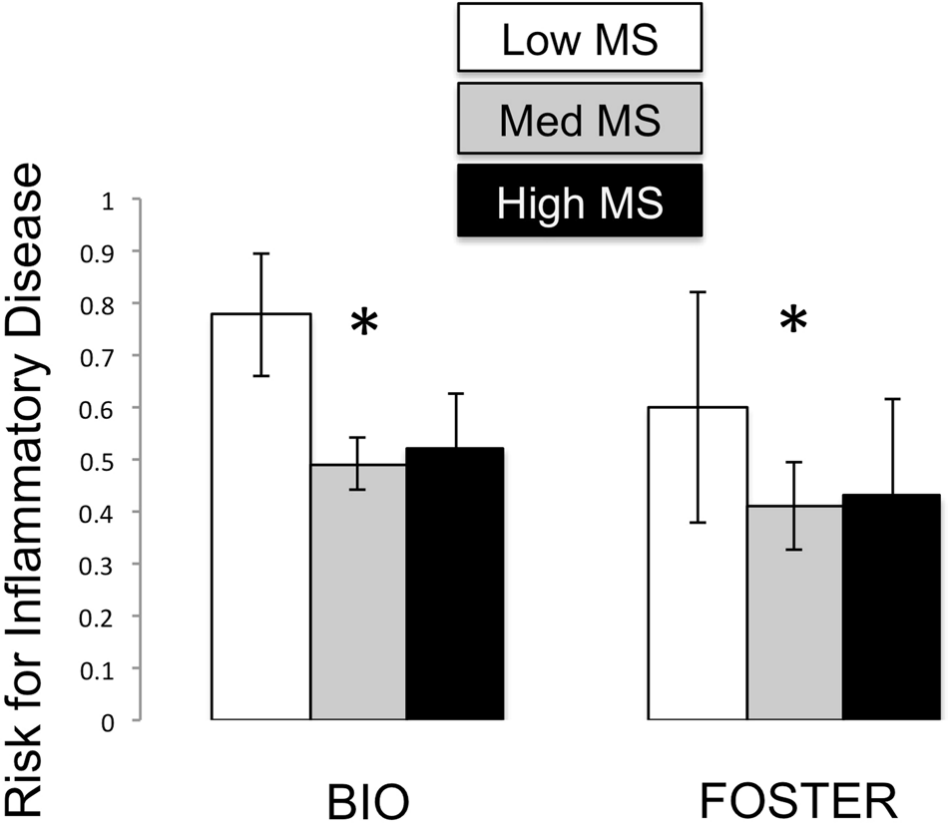
Low MS is associated with a greater risk for disease (colitis/diarrhea) in BIO and FOSTER infants. Means are presented +/− standard error of the mean. *p < 0.05.

Low MS also predicted a greater risk of ever developing diarrhea or colitis up to adulthood, and as late as 12 years of age (chi square = 5.919, p = 0.026; Fig. 2) than medium or high MS. None of the other factors in the model (sex, foster status, maternal social rank or social conflict rates) predicted risk for disease (all p > 0.05). Higher CRP, but not IL-8 or MCP-1, predicted lifetime disease risk (t = 1.995, p = 0.048).

## Discussion

Multiple types of early adversity have been linked with poorer health outcomes across the lifespan. It has recently become clear that inflammation, which results from over-stimulation of the immune system, can link early stress with poor lifespan health^4–11^. We show that a highly translational model of early human stress, low maternal sensitivity (MS), predicts greater inflammatory stress response in the first few months of life, consistent with a study of human infants^44^. Notably, two other types of early social stress, higher group-level conflict and lower maternal rank, additively predicted higher inflammation early in life, independent of MS. While the effects of low MS predicted a greater risk for diarrhea/colitis as the animal aged, consistent with our previous work and the work of others^40,45^, social rank and conflict did not. These data suggest that different types of social stress early in life exerts independent influences on the developing immune system, but the effects of stress in the mother-infant relationship may be particularly persistent in its effects on health across the lifespan.

We present evidence for the long-term and specific influence of a highly translational measure of early stress, low MS, in rhesus monkeys. Our discovery of a naturally occurring maternal behavior dimension that mimics human MS is consistent with a previous study in rhesus monkeys and our own previous work^18,42^. We believe this MS factor models human MS because (1) it incorporates symmetrical responses (ie, the mother matches her response type with the infant overture type), which appear to be more sensitive to the type of transaction the infant “wants”, (2) it negatively loads interfering responses, like “protectiveness”, which limits the infant’s range of movement, (3) it reflects availability of the mother, as all transaction types include maintaining proximity upon infant approach, and (4) it represents acceptance of infant behavior in that mothers react neutrally or affiliatively rather than aggressively or with rejection. There are some notable differences between macaque MS and human MS measures, however. For example, our MS measure did not negatively load rejecting responses as one of the four human dimensions does. Another limitation is that our model does not mimic human MS in its four-dimension structure. Nevertheless, we theorize that low MS is stressful for the infant macaque, as in humans, because it teaches the infant that his/her actions do not elicit predictable responses from the mother and his/her needs may not be promptly met^27–29^.

Our finding that low MS, but not other aspects of maternal care, predicted higher levels of plasma pro-inflammatory cytokines and proteins (CRP, IL-8 and MCP-1) in infancy was consistent with a human and animal literature that shows that different types of early stress, including low MS, are linked with inflammation and poorer health^6–16,20,24^. This finding is notable because it shows that even subtle variation in an infant’s earliest experiences can shift their immune function. Our immune function measure likely reflects the degree to which inflammation responds to stress, rather than baseline inflammation, because our plasma samples were collected two hours after a separation from mothers and social groups and relocation to relatively novel indoor temporary housing. Thus, our results intriguingly suggest that MS shifts animals’ inflammatory stress response. We theorize that low MS, like other stressors^10,11,24^, shifts neuroendocrine-immune communication, resulting in over-stimulation of the immune system during stress. Over time, it is this exaggerated response to stress that my lead to desensitized immune function, uncontrolled inflammation, and tissue remodeling that promotes infectious, neurodegenerative, metabolic and cardiovascular disease^3,9–11,24^. This is supported by our finding that low MS also increased risk for developing one type of disease: diarrhea/colitis. The implication may be that early dysregulation of the immune system sets in motion a set of trajectories that include inflammation and disease, or alternatively, that early inflammation and disease risk are linked. We did not observe correlations between inflammation markers in infancy and risk for colitis, but future studies will systematically gauge this possibility.

Shared genes and shared phenotypes between mother and infant can confound studies of early life environmental programming. We considered the possibility that maternal care effects on infant outcomes could be attributable to maternal care, or to genes that are shared between mother and infant that are linked with maternal behavior and offspring phenotypes. We were able to rule out shared genetic background as a confound of MS because FOSTER infants, which do not share genes with the mother that raised them, showed the same response to low MS as infants reared by their biological mothers. There was no significant statistical interaction between FOSTER status and MS that would suggest a stronger effect in biological dyads. This is an important advance in our understanding of the role of MS in health because offspring development in the presence of low MS can depend on shared environment, shared genes, or both^36,46^. Thus, our study provides support for a true effect of MS rather than genetic confounds on infant inflammation and lifespan health.

We compared the effects of low MS with two other potential social stressors: maternal social rank in infancy and social conflict rates. As predicted, lower maternal rank and greater rates of social conflict predicted greater inflammation in our infants. Current social rank has previously been linked with biomarkers of inflammation in this species^47,48^, but here we show that maternal rank predicts inflammation in offspring before they acquire their own social rank. This is somewhat consistent with the human literature that shows that elements of social life that restrict access to resources and upward mobility (like low socioeconomic status) increases inflammation^6,11^.

Similarly, rates of conflict within the larger social group, in which our subjects may have played central, peripheral, or no role, increased inflammation. This is consistent with human studies that have shown that environmental violence in the neighborhood^49^ or in wartime combat^50^ increases inflammation, and suggests that these more indirect social factors impact the developing immune system at a very young age. Importantly, these factors did not exert their influence through maternal behavior: there were no interactions among MS, rank, and conflict on inflammatory cytokine and protein concentrations. Unlike low MS, however, these stressors, did not impact long-term disease risk. In fact, immune effects of social rank in primates have previously been shown to be somewhat transient: experimentally shifting rank concomitantly shifts immune function, and vestiges of prior ranks are less evident^47^. Less is known about the permanence of impact of social conflict on health, but, like maternal rank, the current conditions may change significantly over the lifetime and became obscured by this or other life factors (eg, chronic illness, additional social stress).

Some human and macaque studies have shown that the health effects of early life stress differ between males and females^5,12,^^51^. There were no significant differences in inflammation or disease between males and females in our study. There were also no interactions between sex and MS, indicating that males and females respond similarly to variation in MS.

The major limitation of this study is that we cannot truly assign causality in the effects of low MS because we did not experimentally manipulate maternal care. It is possible that it is not MS, but some correlate of low MS, that truly predicted infant inflammation. We did not measure nutritional status or disease burden as possible correlates of low MS, for example. By comparing the effects of low MS with other aspects of maternal behavior in our early analyses, however, we did observe that low MS alone predicted inflammation in infancy. Future studies will examine if this effect persists into adolescence and adulthood.

When maternal factors impact infant development, there are larger contextual factors to consider, including gene-environment correlations and related aspects of the social environment. We are able to account for these contextual factors and provide compelling evidence that the type of care an infant receives impacts inflammation and lifespan risk for one common macaque disease, independent of shared genetic background. Importantly, we show that other aspects of the social environment (lower social rank and group-level social conflict) contribute additive and independent variation to early inflammation. We conclude that social stressors may be a key factor in the development of chronic inflammation in infancy, but that stress in the attachment relationship may have a longer-term reach on health outcomes, perhaps due to the uniqueness of the parent-offspring relationship in ontogeny^1,16–18^.
